# Supplementary material for: Circulating Biomarkers of Fibrosis Formation in Patients with Arrhythmogenic Cardiomyopathy
Source: Biomedicines. 2023 Mar 7;11(3):813. doi: 10.3390/biomedicines11030813 (PMC10045011; doi:10.3390/biomedicines11030813)
Supplement: Supplementary file 1 [file biomedicines-11-00813-s001.zip › biomedicines-2251589-supplementary.pdf]

Supplementary Materials

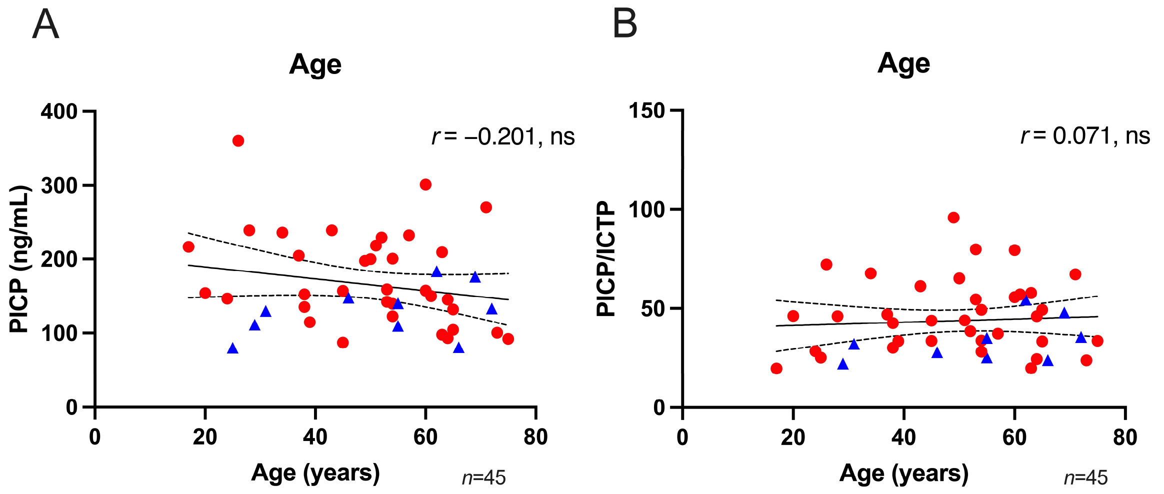

Supplementary Figure S1. No effect of age on biomarker levels. (**A**) A weak negative correlation of age with PICP levels was seen (*n*=45). (**B**) No correlation of total collagen turnover and age was found (*n*=45). Blue triangles represent preclinical variant carriers, while red dots are affected ACM patients. PICP; procollagen type I carboxy-terminal propeptide, ICTP; C-terminal telopeptide collagen type I, *r*; Pearson correlation coefficient, ns; not significant. Pearson's correlation coefficient was performed.

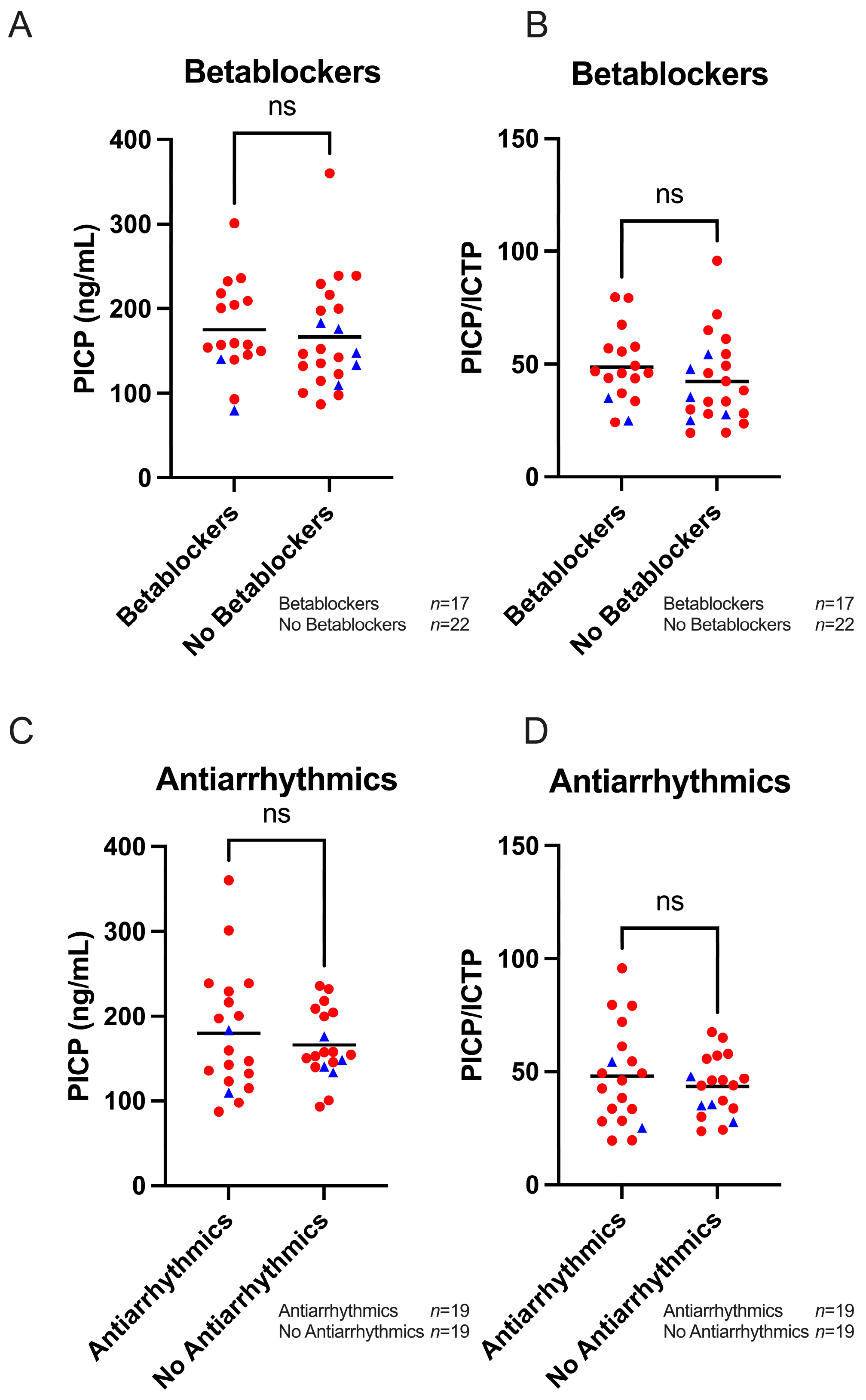
A
Betablockers
ns
400
300
200
100
0
PICP (ng/mL)
Betablockers
No Betablockers
Betablockers n=17
No Betablockers n=22
B
Betablockers
ns
150
100
50
0
PICP/ICTP
Betablockers
No Betablockers
Betablockers n=17
No Betablockers n=22
C
Antiarrhythmics
ns
400
300
200
100
0
PICP (ng/mL)
Antiarrhythmics
No Antiarrhythmics
Antiarrhythmics n=19
No Antiarrhythmics n=19
D
Antiarrhythmics
ns
150
100
50
0
PICP/ICTP
Antiarrhythmics
No Antiarrhythmics
Antiarrhythmics n=19
No Antiarrhythmics n=19

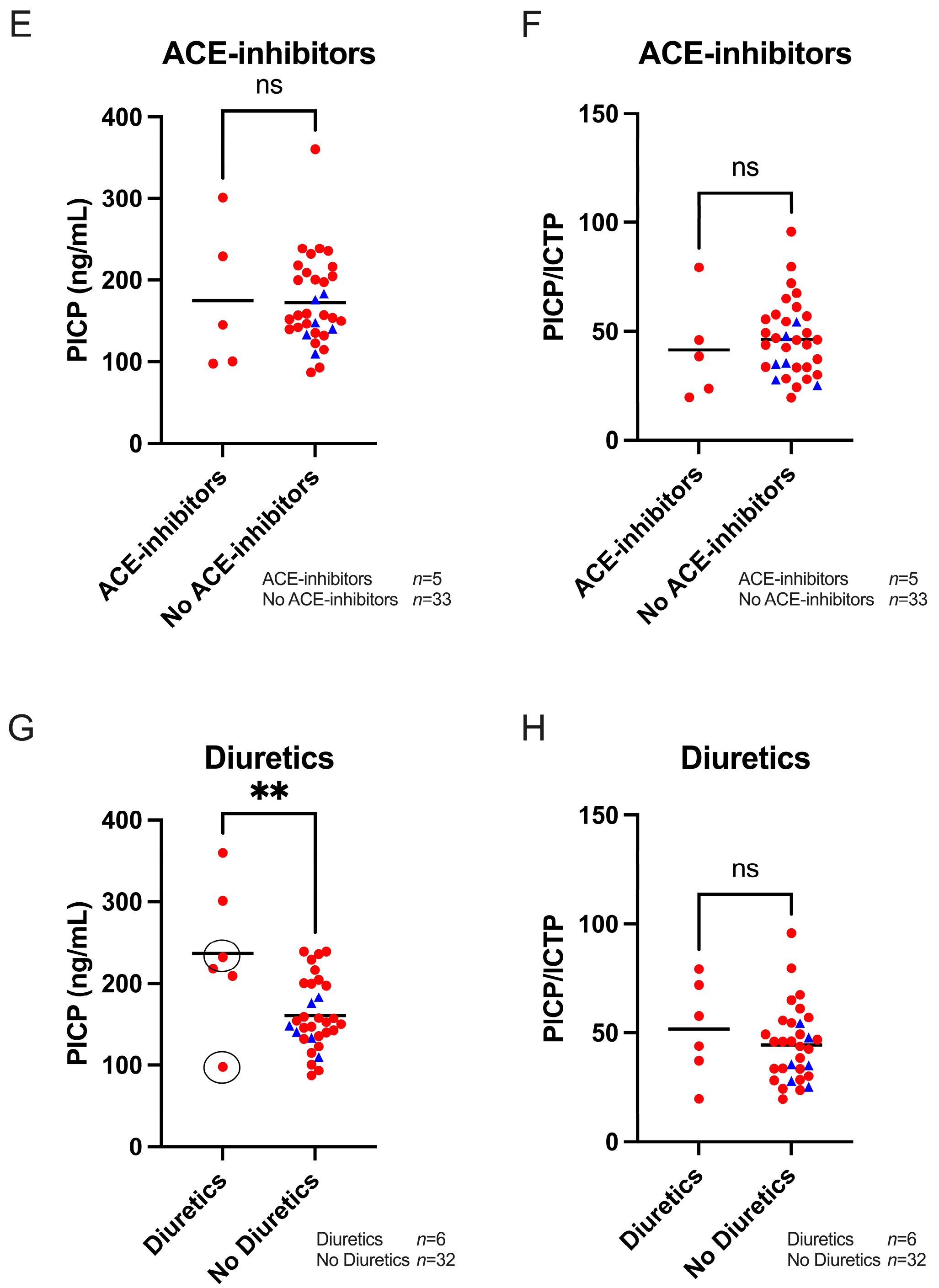

Supplementary Figure S2. Medication use did not influence fibrosis biomarker level. (**A**,**B**) The use of betablockers (*n*=17) did not influence PICP and PICP/ICTP ratios compared to patients without betablockers (*n*=22). (**C**,**D**) Fibrosis biomarker levels were not changed when using antiarrhythmics (*n*=19 both groups). (**E**,**F**) Fibrosis biomarkers levels were similar between patients using ACE-inhibitors (*n*=5) and patients without using ACE-inhibitors (*n*=33). (**G**) Significant higher levels of PICP levels were found in patients using diuretics (*n*=6). Marked patients correspond to patients using torasemid as diuretics. (**H**) Similar levels of total collagen turnover were found in patients with (*n*=6) and without using diuretics (*n*=32). Blue triangles represent preclinical variant carriers, while red dots are affected ACM patients. PICP; procollagen type I carboxy-terminal pro-peptide, ICTP; C-terminal telopeptide collagen type I, ACE; angiotensin-coverting enzyme, ns; not significant. Unpaired Student's t test is performed. $^{**}p<0.01$.

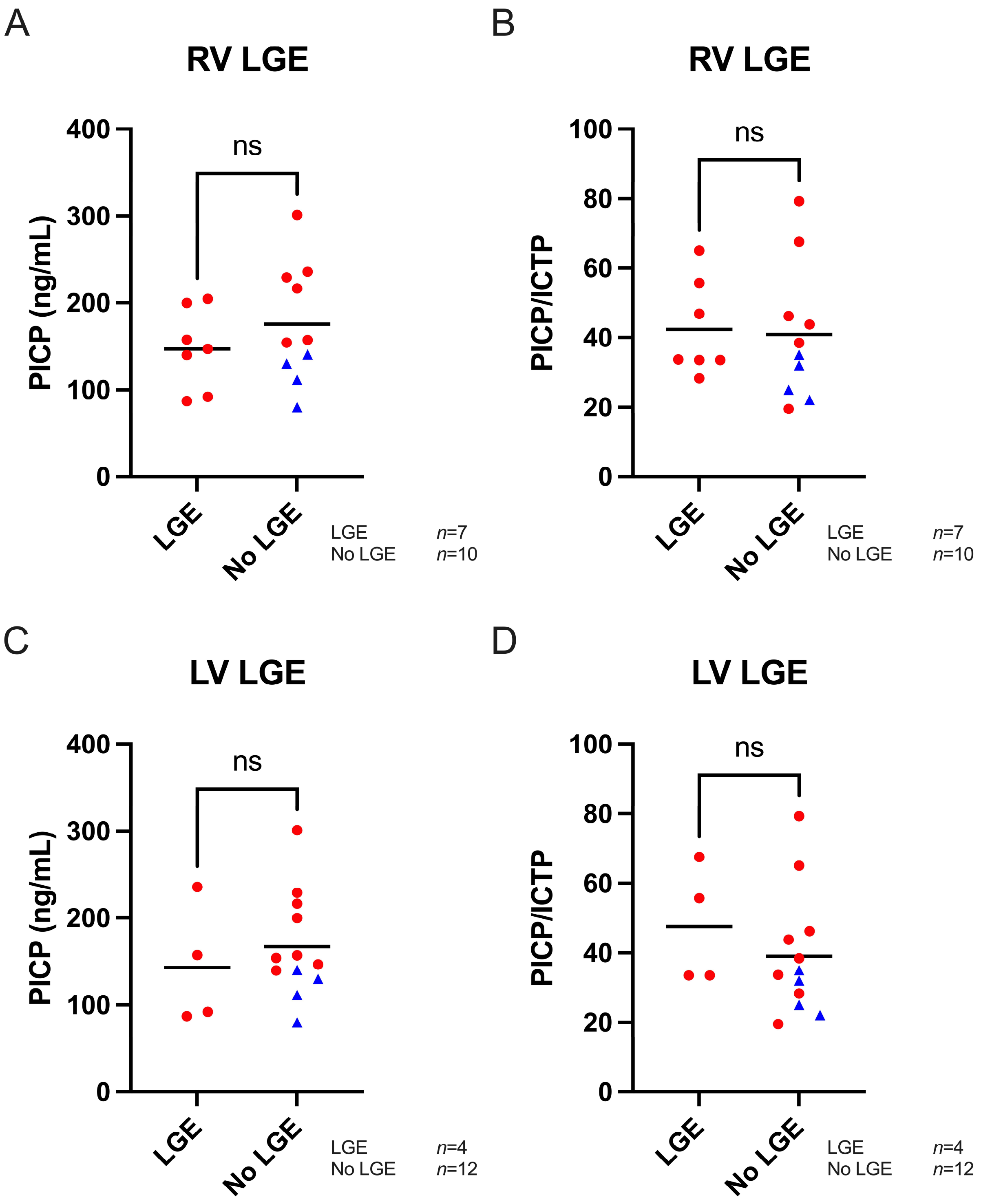

Supplementary Figure S3. No correlation with LGE and fibrosis biomarkers. (**A**,**B**) No difference in PICP levels or PICP/ICTP ratio in ACM patients with RV LGE (*n*=7) compared to patients without RV LGE (*n*=10). (**C**,**D**) No significant distinction was found between patients with LV LGE (*n*=4) and patients without LV LGE (*n*=12) and fibrosis biomarkers. Blue triangles represent preclinical variant carriers, while red dots are affected ACM patients. PICP; procollagen type I carboxy-terminal pro-peptide, ICTP; C-terminal telopeptide collagen type I, LGE; late gadolinium enhancement, RV; right ventricle, LV; left ventricle, ns; not significant. Unpaired Student's t test was performed.
